# Limited angular remodelling after in-situ fixation for slipped capital femoral epiphysis: A study on radiographs from the Swedish pediatric orthopaedic quality registry for SCFE

**DOI:** 10.1186/s12891-023-07117-y

**Published:** 2024-01-02

**Authors:** Mattias Anderson, Bengt Herngren, Hans Tropp, Olof Risto

**Affiliations:** 1 Department of Orthopaedics, University Hospital, Linköping, 58185 Sweden; 2 Department of Biomedical and Clinical Sciences, Linköping University, Linköping, Sweden; 3 Futurum – Academy for Health and Care, Jönköping county council, Department of Orthopaedics, Ryhov county hospital, Jönköping, Sweden; 4 Centre for Medical Image Science and Visualization (CMIV), Linköping University, Linköping, Sweden

**Keywords:** Slipped capital femoral epiphysis, SCFE, Remodelling

## Abstract

**Background:**

In Sweden, most children with slipped capital femoral epiphysis (SCFE) are operated on with a single smooth pin or a short-threaded screw, allowing further growth of the femoral neck. Using the Swedish Pediatric Orthopaedic Quality registry, SPOQ, we investigated whether angular remodelling occurs adjacent to the proximal femoral epiphysis after fixation of SCFE using implants, allowing continued growth of the femoral neck.

**Methods:**

During 2008–2010 a total national population of 155 children were reported to the SPOQ registry. Following our strict inclusion criteria, radiographs of 51 hips were further assessed. The lateral Head Shaft Angle (HSA), the Nötzli 3-point α-angle, the anatomic α-angle, and the Anterior Offset Ratio (AOR) on the first postoperative radiographs and at follow-up were measured to describe the occurrence of remodelling. Slip severity was categorised as mild, moderate or severe according to postoperative HSA.

**Results:**

Mean and SD values for the change in HSA were 3,7° (5,0°), for 3-point α-angle 6,8° (8,9°), and anatomic α-angle 13,0° (16,3°). The overall increase in AOR was 0,038 (0.069). There were no significant differences between the slip severity groups.

**Conclusions:**

We found limited angular remodelling after in situ fixation with smooth pins or short threaded screws for SCFE. The angular remodelling and the reduction of the CAM deformity was less than previously described after fixation of SCFE with similar implants. Results about the same magnitude with non-growth sparing techniques suggest that factors other than longitudinal growth of the femoral neck are important for angular remodelling.

## Introduction

Slipped Capital Femoral Epiphysis (SCFE) is Sweden´s most common adolescent hip disease, with an incidence rate of 5 per 10 000 children aged 9–15 [1]. In SCFE, the metaphysis slips in relation to the proximal femoral physis. With increasing slip angles, the anterosuperior head-neck junction will be more convex than the normal concave shape. This prominent shape of the femoral neck metaphysis, called CAM-deformity, increases the risk of symptomatic femuro-acetabular impingement (FAI) [2]. FAI is a mechanical conflict between the head-neck junction and the acetabular rim with hip flexion resulting in subsequent damage to the labrum and the acetabular cartilage [3].

With FAI after SCFE, the cartilage damage is seen early, and the severity seems worse in hips with intermediate slip angles [4].

**Fig. 1:**
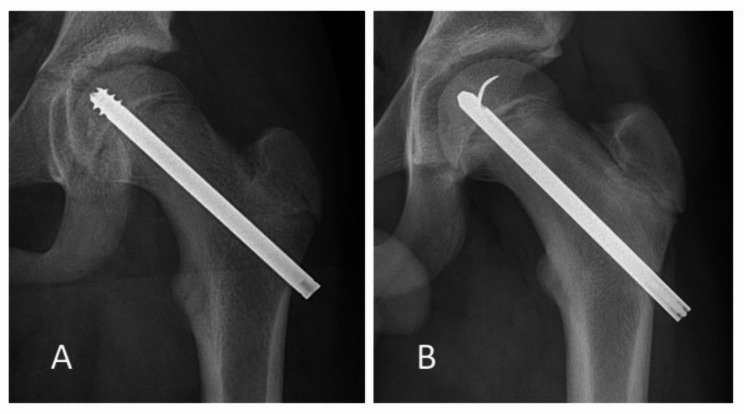
Implants included in the study. (**A**) Short-threaded screw (Olmed) (**B**) Hansson hook pin

Surgery with fixation in situ is the most commonly used method to prevent slip progress and minimise the CAM-prominence after SCFE. Generally, a percutaneous screw with threads crossing the physis to achieve epiphysiodesis is used. However, epiphysiodesis might cause abnormal hip anatomy. In addition, with a CAM deformity, the short neck might interfere with the hip’s normal mechanics [5]. Therefore, most children in Sweden with SCFE are operated upon with a single smooth pin or a short-threaded screw that reliably prevents slip progress and allows continued growth of the femoral neck [6–8] (Fig. 1).

Remodelling of bone is a complex process to repair and replace old bone and regain a more normal shape of the affected bone [9]. Remodelling is suggested to occur after in situ fixation of SCFE regardless of the surgical method used [10–12]. The early phase of SCFE remodelling occurs by osteoclastic resorption of the anterosuperior femoral metaphysis and callus formation at the inferior-posterior angle between the head and neck [13, 14].

**Fig. 2:**
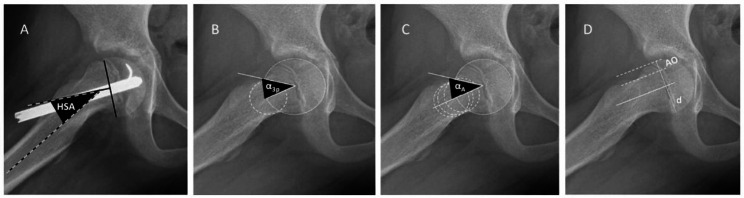
(**A**) The lateral head shaft angle (HSA) is the angle between the femoral shaft axis and a line perpendicular to a line connecting the femoral head’s physeal edges. (**B**) The 3-point α-angle method as defined by Nötzli (α_3p_). Place a circle over the femoral head’s bony contour and another circle over the femoral neck’s narrowest part. Draw a line connecting the centre of these two circles. Then draw a line from the femoral head’s centre to where the head-neck contour exits the femoral head circle. The α_3p_ is the angle between these two lines. (**C**) The anatomic α-angle (α_A_) as described by Bouma. Determine the axis of the femoral neck by placing three circles touching the contour of the femoral neck. Draw a line through the centres of the neck circles to decide the anatomic femoral neck axis. The neck axis is translated to the centre point in the best-fit circle over the femoral head. A second line is drawn from the central point to where the head-neck contour first exits the femoral head circle. The α_A_ is the angle between these two lines. (**D**) The anterior offset ratio. First, draw a line through the centre of the femoral neck. Then two parallel lines are drawn along the anterior edge of the neck and the femoral head. The distance between the latter (AO) divided by the femoral head diameter (d) gives the anterior offset ratio (AOR), also called the head-neck offset ratio (HNOR)

The lateral head shaft angle (HSA), as defined by Southwick [15], is the most common way to measure epiphyseal angulation (Fig. 2). Measures to quantify the CAM deformity include the anterior offset ratio (AOR) [16, 17] and the α-angle (Fig. 2). Although first described by Nötzli [18] on radial magnetic resonance imaging, the α-angle is now commonly measured on lateral radiographs [19, 20], as is the AOR [21]. Two ways for measuring the α-angle are described, the 3-point method (α_3p_) as defined by Nötzli [18, 22, 23] and the anatomic method (α_A_) described by Bouma [24]. In a normal hip, α_3p_ will equal the α_A_.

A mean angular remodelling of 9 degrees using a single smooth pin was recently reported [19]. In addition, other European studies with similar implants suggest that angular remodelling does occur [25, 26].

There is no consensus on whether remodelling will alter the shape of the hip after fixation in situ and thereby prevent FAI symptoms. New treatment algorithms, including safe surgical dislocation with capital realignment, are gaining popularity [27–30].

The present study aimed to investigate whether angular remodelling, with a subsequent reduction of CAM-deformity, occurs adjacent to the proximal femoral epiphysis after fixation of SCFE using an implant allowing continued growth of the femoral neck. Data from the Swedish Pediatric Orthopaedic Quality (www.spoq.se) registry was used.

## Methods

This study is based on the radiographs of all children (155 children, 171 hips) consecutively reported to the Swedish Pediatric Orthopaedic Quality (SPOQ) registry, 2008–2010. The registry compared its database with individual-based data from the Swedish National Board of Health and Welfare, utilising the Swedish National Patient Register. The study population included a total national population for the study period. Thus, the first author assessed the radiographs of all persons who had surgery because of SCFE in Sweden during these three years (MA). Each participant consented to inclusion in the Swedish Pediatric Orthopaedic Quality registry.

The radiographs were routine examinations from all Swedish hospitals that treated children with SCFE during the study period. Postoperative and follow-up radiographs were assessed using the standard picture archiving and communication system (IDS7, Sectra, Linköping, Sweden).

Inclusion criteria were SCFE surgery performed with one smooth hook-pin or one smooth short-threaded screw and frog-leg lateral (Lauenstein) postoperative and follow-up radiographs with at least eight months intervals. For each hip, we used radiograph pairing to identify unacceptable rotational differences between the postoperative and follow-up radiographs. The Southwick head shaft angle (HSA) assessment required no less than 2 cm of the femoral shaft visible [31]. To rule the radiographs as comparable or not, apart from the shaft length, was a judgement based on a combination of the following criteria: we did not accept a variance in the visible amount of the lesser trochanter, the direction of the hook of the Hansson hook pin, or other differences in the implant position.

Thus, radiographs with inconsistent quality or lateral view other than Lauenstein, surgery performed with other methods, primary intentional physeodesis, subsequent early surgery performed (e.g., proximal femoral osteotomy) or development of avascular necrosis were excluded.

Following our strict inclusion criteria, the study material included radiographs of 51 hips. Each hip was given a unique number, thus blinding the observer to age and name. The first author assessed postoperative and follow-up radiographs twice, with a minimum interval of one month. The first measurement findings were unavailable for the observer during the second measurement. Therefore, each hip was presented with the mean of the two measurements. The baseline measurements were done on the first postoperative radiograph. All follow-up radiographs had signs of finished growth at the femoral neck and, importantly, were of the same frog-leg lateral projection as the immediate postoperative control.

The lateral head-shaft angle (HSA) was used as defined by Southwick [15]. The slip severity was classified according to the baseline HSA (mild < 30°, moderate 30–60°, and severe > 60°) [12, 19]. The AOR and lateral offset angle (α-angle) describe the CAM deformity of the head-neck junction. The anatomic method (α_A_) described by Bouma [24] was compared with the 3-point method (α_3p_) as defined by Nötzli [18, 22, 23] (Fig. 2).

When comparing slip severity groups according to baseline HSA, the hips excluded from further study were not significantly different from those included for further research. Slip severity at baseline HSA for the excluded group was comparable with the group included in the study: mild (mean HSA 19,0°), moderate (mean HSA 42,0°) and severe (mean HSA 66,0°). Median age at surgery was 12.4 years in the excluded group and 12.5 years in the study group. We believe the study group represents the whole group of patients.

### Statistics

Results are presented as mean values with range or standard deviation. We set the significance threshold at 0.05. The Student t-test (independent samples) was used to compare means for postoperative and follow-up variables for gender and age groups. The paired sample t-test was used to compare the means of postoperative and follow-up variables. The Pearson correlation coefficient was used to test the correlation between HSA, the two alpha angle methods, HSA at follow-up, AOR, and age at surgery. ANOVA with post hoc Tukey test was used to compare variables with the groups of different slip severity. Intra-rater reliability (ICC) and 95% confidence interval (CI) are based on an absolute-agreement, 2-way mixed-effects model for single measures. Variation of intraobserver measurements was also presented as Bland-Altman plots. The statistical analysis was performed using SPSS Statistics (version 25; IBM, Armonk, NY).

## Results

Matching radiographs of good quality were found in 51 hips (48 children; 3 bilateral). The fixation method used was one smooth hook-pin (n = 28) or one smooth short-threaded screw (n = 23). The type of implant did not significantly affect the measured variables.

We studied radiographs of 18 boys and 30 girls with a median age of 12.5 years at surgery, girls 11.6 years (range; 9.5–14.6) and boys 13.9 years (range; 11.2–14.6) (*p* < 0,001). The median interval between postoperative and follow-up radiographs was 25.2 months (range; 9.6–68.4). Except for age at surgery, we found no statistical differences between boys and girls regarding HSA, α_3A_, α_3p_, or AOR at baseline.

Between the baseline and follow-up radiographs, the mean reduction of HSA was 3.7° (SD 5°), the mean decrease in α_3p_ was 6.8° (SD 8.9°), the mean reduction of α_A_ was 13° (SD 16.3°), and the mean increase of the AOR was 0,038 (SD 0.069). The differences found were statistically significant (*p* < 0.001). Table 1.

**Table 1:** Characteristics and results for the study population of 51 hips

	Mean	SD	CI(95%)	*P*-value
Age at surgery (years)	12.3	1.65		
Follow-up time (months)	28.5	13.9		
Postoperative HSA (baseline HSA)	38,5°	17,4		
Follow-up HSA	34,8°	16,8		
Reduction of HSA	3,7°	5,0	2,5–5,1°	< 0.001
Postoperative α_3p_	67,1°	14,0		
Follow-up α_3p_	60,2°	14,2		
Reduction of α_3p_	6,8°	8,9	4,3–9,3°	< 0.001
Postoperative α_A_	81,7°	21,6		
Follow-up α_A_	68,7°	22,9		
Reduction of α_A_	13,0°	16,3	8,4–17,6°	< 0.001
Postoperative AOR	0,32	0,135		
Follow-up AOR	0,358	0,144		
Increase of AOR	0,038	0,069	0,019 − 0,057	< 0.001

**Fig. 3:**
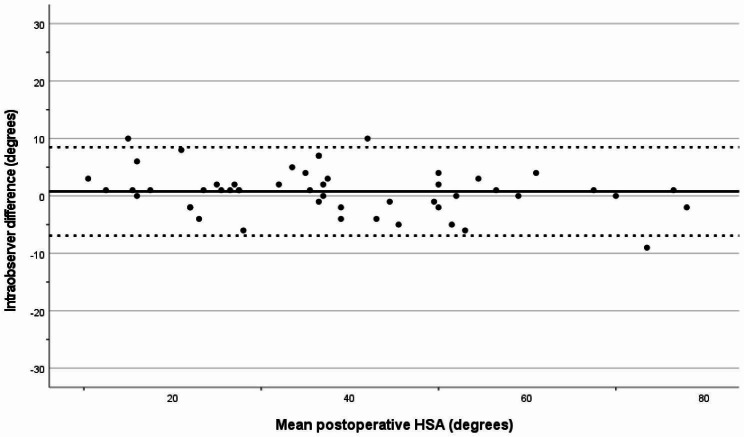
Intraobserver variation (degrees) for postoperative HSA. The solid line represents the mean value and the dotted lines show the limits of agreement (mean +/- 1.96SD)

**Fig. 4:**
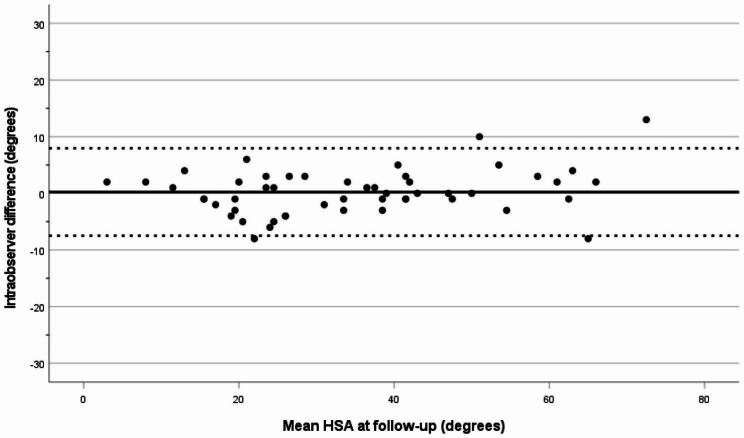
Intraobserver variation (degrees) for HSA at follow-up. The solid line represents the mean value and the dotted lines show the limits of agreement (mean +/- 1.96SD)

Intra-observer variation for the HSA measurements was assessed using the mean difference, with its 95% limits of agreement [31, 32]. For the postoperative HSA, the intra-observer difference was 0.8°, and for the HSA at follow-up the difference was 0.2° (Figs. 3 and 4). The intra-observer reliability for the HSA was excellent, with an ICC of 0.97 (CI 0.95–0.98).

**Fig. 5:**
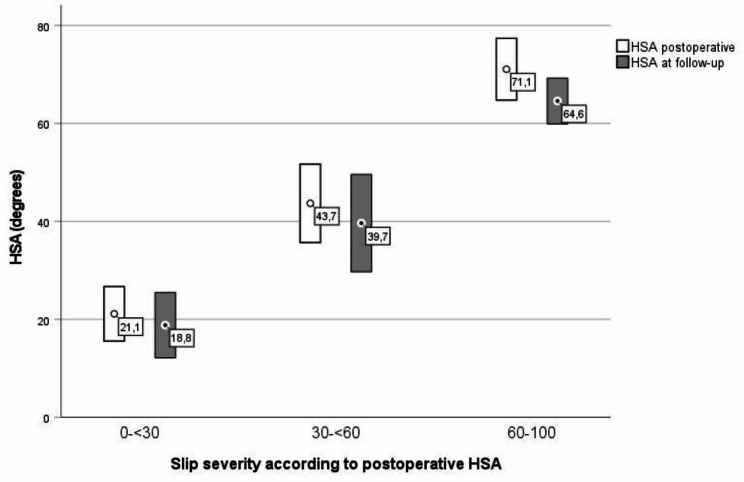
Postoperative and follow-up HSA according to slip severity at baseline (mean ± SD)

According to baseline HSA, there were 19 mild (mean HSA 21,1°), 26 moderate (mean HSA 43,7°) and six severe (mean HSA 71,1°) slips. The mean HSA did decrease during growth, but it did not differ significantly between the groups of severity (Fig. 5).

The difference between the three groups of slip severity concerning HSA, α_A_, and AOR was statistically significant at baseline. However, for the postoperative α_3p_, the difference between the moderate and severe groups of slip severity was not statistically significant. There were no significant differences between the slip severity groups regarding reducing HSA, α_3p_, α_A_, or the increase of AOR (Table 2).

**Table 2:** Characteristics and results according to slip severity (mean ± SD)

	<30° (N = 19)	30-<60° (N = 26)	>60° (N = 6)
Age at surgery (years)	11,8	12,5	12,9
Reduction of HSA	2,3° (4,0)	4,0° (5,4)	6,5° (5,3)
Reduction of α_3p_	6,2° (8,4)	8,1° (9,1)	3,2° (9,4)
Reduction of α_A_	13,1° (15,7)	14,4° (17,6)	6,5° (12,8)
Increase of AOR	0,04 (0,07)	0,04 (0,07)	0,01 (0,07)

**Fig. 6:**
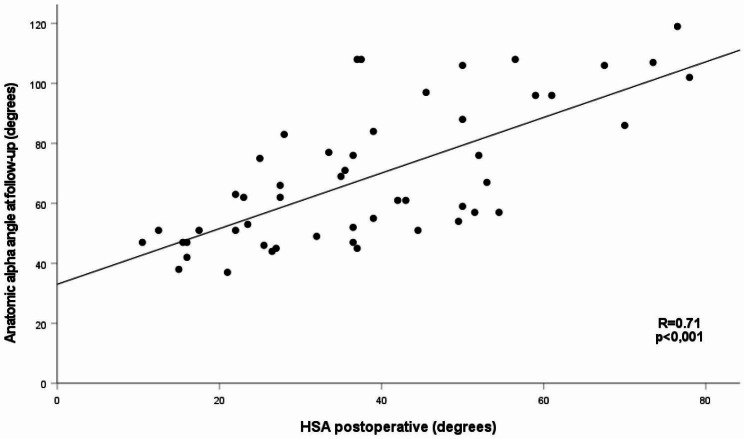
The correlation between postoperative HSA and the α_A_ at follow-up

There was a significant inverse correlation between the postoperative HSA and AOR at follow-up (R=-0.69, *p* < 0.001) as well as a significant correlation between postoperative HSA and both methods to measure the α-angle at follow-up (α_3p_; R = 0,68 and α_A;_ R = 0,71, *p* < 0.001), (Fig. 6).

**Fig. 7:**
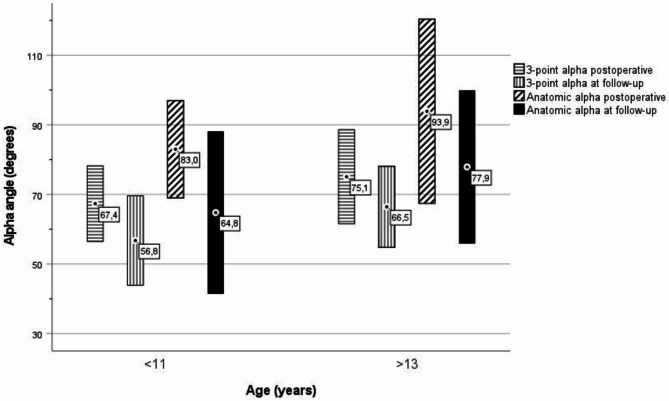
Postoperative and follow-up α-angle (α_3p_ and α_A_) in children younger than 11 and older than 13 years at surgery (mean +/-SD)

Fourteen children were ten years of age or younger, and 11 children were 14 years of age or older at surgery. The mean follow-up time between the age groups differed significantly. The mean HSA at baseline was higher in the older group (34,6° and 47,3°, respectively) (*p* = 0,07). At follow-up, the mean HSA was significantly lower in the younger age group (28,9° and 42,5°, respectively) (*p* = 0,05). The reduction of HSA, α_3p,_ α_A,_ and the increase of AOR was larger for the younger group but not statistically significant. (Table 3; Fig. 7).

**Table 3:** Results in children younger than 11 and older than 13 years at surgery (mean +/-SD)

Age at surgery (years)	< 11(n = 14)	> 13 (n = 11)	*P*-value
Follow-up time (months)	36,0 (15,2)	22,8 (7,2)	0.015
HSA postoperative (degrees)	34,6 (16,4)	47,3 (16,8)	0.70
HSA follow-up (degrees)	28,9 (17,2)	42,5 (14,9)	0.05
Reduction of HSA (degrees)	5,6 (4,6)	4,7 (5,8)	0.68
α_3p_ postoperative (degrees)	67,4 (10,9)	75,1 (13,5)	0.13
α_3p_ follow-up (degrees)	56,8 (12,8)	66,5 (11,6)	0.06
Reduction of α_3p_ (degrees)	10,8 (12,7)	8,6 (6,3)	0.62
α_A_ postoperative (degrees)	83,0 (14,0)	93,9 (26,5)	0.24
α_A_ follow-up (degrees)	64,8 (23,3)	77,9 (22,0)	0.17
Reduction of α_A_ (degrees)	18,2 (19,0)	16,0 (15,1)	0.76
AOR postoperative	0,347 (0.095)	0,226 (0.216)	0.11
AOR follow-up	0,400 (0.089)	0,252 (0.233)	0.07
AOR increase	0,054 (0.060)	0,027 (0.077)	0.34

## Discussion

Compared to other studies of surgery for SCFE with growth-sparing techniques, we found statistically significant (*p* < 0.001) but only limited angular remodelling. Wensaas and Svenningsen found an average HSA remodelling of 15°, Guzzanti et al. found 13,5°, and Örtegren et al. found 9° compared to our 3,7° [19, 25, 26]. Our strict inclusion criteria, for eligible hips to be assessed, might explain this difference.

Unlike Örtegren et al. [19], we did not find statistically significant differences regarding the reduction of HSA between the different slip severity groups. In the study by Örtegren et al., only three hips were included in the severe group showing a mean HSA reduction of as much as 31,3°, whereas our six severe hips remodelled by a mean of 6,5°.

We don´t believe remodelling is clinically important for the group with a mild slip. The group with a severe slip has a high risk for symptomatic FAI, with a remodelling to a mean of 64,6° HSA. Whether the slight mean HSA decrease of 4° in the moderate group might reduce the risk for symptomatic FAI is unclear but unlikely. Accabled et al. found a significant correlation between slip severity and patient-reported outcome showing that HSA values exceeding 35° gave increased risk for symptomatic FAI [33]. Also, Nectoux et al. and Murgier et al. have pointed out the threshold of slip angles of 30–35° for symptomatic FAI to appear more regularly [34, 35].

The importance of continued longitudinal growth of the femoral neck for angular remodelling and, thereby, a decreased risk for symptomatic FAI is supported by Örtegren et al. [19]. With a growth-sparing technique, the femoral neck growth continues with 3–4 mm/year [7] to a total of 8,9–15,2 mm [8]. We did not measure the longitudinal growth of the femoral neck. However, angular remodelling about the same magnitude with non-growth sparing techniques suggests other factors’ importance [11, 12].

Remodelling depends on the remaining growth potential. Örtegren et al. found a statistically significant difference in HSA reduction between children younger than 11 years of age and children older than 13 [19]. We did the same analysis with the finding of a more minor and statistically not significant difference. Regardless of the age at surgery, we found strong correlations between HSA postoperatively and α_3p_, α_A_, AOR, and the HSA at follow-up. These findings support the importance of factors other than femoral neck growth for angular remodelling.

Our study showed a statistically significant difference between the severity groups at baseline regarding α_A_ and AOR but not for the α_3p_. The better correlation at follow-up between AOR and α_A,_ than for α_3p,_ suggests that α_A_ corresponds better to the anterior head-neck offset, as pointed out in other reports [24, 36].

We found that assessing the morphology of the SCFE hip was simplified using the anatomical alpha angle rather than the 3-point method. The α_A_ correlates better with the AOR as both methods reference the femoral neck axis. For hips with poor head-neck offset, i.e. higher HSA, the alpha angle will increase accordingly. Therefore, HSA and the α_A_ probably better judge the risk of symptomatic FAI connected to SCFE than the α_3p_ does, which is also pointed out by Ucpunar et al. (39).

The gender distribution in our study included a higher percentage of girls than boys compared to the population-based study by Herngren et al. [1]. Therefore, we do not have any reason to believe that the remodelling is different between girls and boys.

### Limitations

The exclusion of 72 hips was because of inconsistent quality and rotational error. The pair-wise inspection of the radiographs to ensure the same visibility of the lesser trochanter and identical implant position on the frog-leg views contributed to this. However, we became confident that we ended up with 51 pairs of radiographs with as small a rotational error as possible.

Another limitation is that only the first author made measurements. However, the first and second measurements were blinded, assessed randomly and made twice, at least one month apart. The excellent intraobserver reliability, with an ICC of 0.97 for the HSA, is a factor that makes us believe we have reliable measurements for the analysis [37].

The setup based only on radiographs available in a registry is a limitation. This retrospective study revealed that clinical practice regarding the type of radiographs and projections varied considerably in Sweden from 2008 to 2010. Many radiographs were of inferior quality and the type of projections used varied with time. The majority of exclusions were made because the correct and comparable measurements was not possible to make. Nevertheless, the study material was based on a total national population in Sweden during the study period chosen.

## Conclusion

We found limited angular remodelling after in situ fixation with smooth pins or short threaded screws for SCFE. The angular remodelling and the reduction of the CAM deformity were less than previously described after the fixation of SCFE with similar implants.

Results about the same magnitude with non-growth sparing techniques suggest that factors other than longitudinal growth of the femoral neck are important for angular remodelling.

## Data Availability

The datasets in this study are available from the corresponding author on reasonable request.

## List of Abbreviations

SCFE: slipped capital femoral epiphysis
SPOQ: Swedish Pediatric Orthopaedic Quality registry
HSA: Head Shaft Angle
α_3p_: 3-point α-angle
α_A_: Anatomic α-angle
AOR: Anterior Offset Ratio
SD: Standard deviation
FAI: Femuro-acetabular impingement
ICC: Intra-rater reliability
